# Crop booms as regime shifts

**DOI:** 10.1098/rsos.231571

**Published:** 2024-06-19

**Authors:** Victoria Junquera, Maja Schlüter, Juan Rocha, Nico Wunderling, Simon A. Levin, Daniel I. Rubenstein, Jean-Christophe Castella, Patrick Meyfroidt

**Affiliations:** ^1^ High Meadows Environmental Institute, Princeton University, Princeton, NJ, USA; ^2^ Department of Ecology and Evolutionary Biology, Princeton University, Princeton, NJ, USA; ^3^ Stockholm Resilience Centre, Stockholm University, Stockholm, Sweden; ^4^ FutureLab Earth Resilience in the Anthropocene, Potsdam Institute for Climate Impact Research (PIK), Potsdam, Germany; ^5^ Center for Critical Computational Studies (C³S), Goethe University Frankfurt, Frankfurt am Main, Germany; ^6^ SENS (IRD, CIRAD, UPVM), Université de Montpellier, Montpellier, France; ^7^ Earth and Life Institute, UCLouvain, Louvain-la-Neuve, Belgium; ^8^ F.R.S.-FNRS, Brussels, Belgium

**Keywords:** crop booms, land regime shifts, self-reinforcing feedbacks, complex systems, Laos, frontiers

## Abstract

A crop boom is a sudden, nonlinear and intense expansion of a new crop. Despite their large impacts, boom-bust dynamics are not well understood; booms are largely unpredictable and difficult to steer once they unfold. Based on the striking resemblances between land regime shifts and crop booms, we apply complex systems theory, highlighting the potential for regime shifts, to provide new insights about crop boom dynamics. We analyse qualitative and quantitative data of rubber and banana plantation expansion in two forest frontier regions of northern Laos. We show that *preconditions*, including previous booms, explain the *occurrence* (why) of booms, and *triggers* like policy and market changes explain their *timing* (when). Yet, the most important features of booms, their *intensity* and nonlinearity (how), strongly depended on internal self-reinforcing *feedbacks*. We identify built-in feedbacks (neighbourhood effects and imitation) and emergent feedbacks (land rush) and show that they were *social* in nature, *multi-scale* from plot to region and subject to thresholds. We suggest that these are regular features of booms and propose a definition and causal-mechanistic explanation of crop booms, examining the overlap between booms and regime shifts and the role of frontiers. We then identify opportunities for management interventions before, during and after booms.

## 1. Introduction

Crop booms are a form of agricultural expansion characterized by their abruptness, speed, intensity and nonlinearity [1]. Notable instances include booms of oil palm, cocoa and rubber in Southeast Asia; coffee in Vietnam; cocoa in West Africa; and soybean in South America [2–6]. All of these have occurred in natural resource frontiers—where land and natural resources are abundant but labour and capital are initially scarce [7]—as farmers or agribusiness corporations converted natural or extensively used ecosystems to commercial (cash) crop plantations. Alternatively, crop booms can take place in long-standing intensive agricultural landscapes when a commercial crop replaces others, as in the case of *Honeycrisp* apple plantations in Nova Scotia, Canada [8] or the switch from citrus to avocado in Valencia, Spain [9].

Crop booms tend to exhibit an S-shaped or logistic pattern [10], where a period of slow early adoption is followed by rapid expansion, which precedes a phase of declining growth rates and saturation owing to land scarcity, declining soil quality or other limiting factors. The speed of expansion, combined with the ‘busts’ that frequently follow [11–14], can intensify the social and ecological impacts [15].

Given the ubiquity of booms and their potentially large social and ecological outcomes, it is important to understand the underlying causal mechanisms [1]. Yet, boom-bust dynamics are not well understood, in part because most models and theoretical frameworks are designed to study gradual, continuous land system change rather than abrupt, nonlinear and structural transformations [16–19]. Partly as a result, land governance institutions are generally unable to anticipate [19] or even recognize crop booms early and frequently lack the capacity to steer such transformations towards desirable outcomes [1].

An explanation for the characteristic dynamics of crop booms can be found in the literature on complex systems, innovation adoption and opinion dynamics, which has extensively documented abrupt and rapid changes such as regime shifts, S-shaped adoption patterns or sudden flips in behaviour. All of these nonlinear and threshold responses have been linked to the presence of self-reinforcing feedbacks [20,21], such as imitation [22–25] or a critical mass beyond which a minority opinion takes over [23,26].

**Figure 1.**
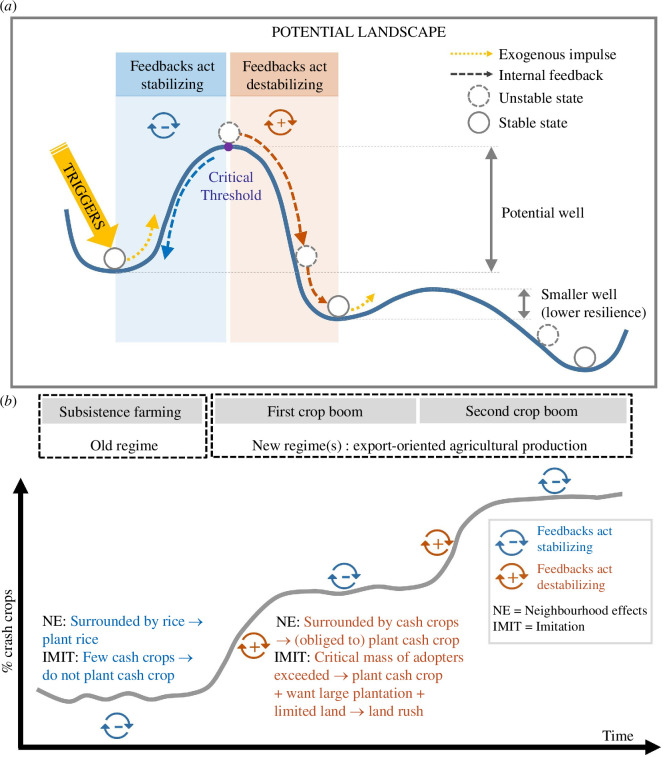
(*a*) One class of regime shifts is frequently illustrated with a diagram of a potential landscape, in which the state of the system is given by the position of a ball rolling on the landscape. Regime shifts may occur through the transformation of the system from one basin of attraction to another (for example, from subsistence farming to cash crops (*b*)) owing to an exogenous perturbation or trigger, which ‘pushes’ the ball beyond a critical threshold—at which feedbacks become destabilizing—from one valley to the next. Alternatively, an erosion of resilience can reduce or eliminate a former basin of attraction and induce a regime shift. The occurrence of previous booms can have such an effect, making it more likely that another boom will occur.

In fact, crop booms exhibit many similarities with regime shifts, which are large, nonlinear and often persistent changes in the structure and function of complex systems—be they ecological [27], social [26] or coupled social-ecological systems [28–30]—from one relatively stable state or dynamic pattern (regime) to another [21,27,31]. Regime shifts generally occur when a system crosses one or more critical thresholds, also known as tipping points, beyond which the internal feedbacks (positive and negative) that normally stabilize the system become destabilizing. This sets off cascading reactions [32], causes a nonlinear transformation that fundamentally changes the system’s dynamics, characteristics and feedbacks [33] and is closely related to what René Thom termed a ‘catastrophe’ [34]. Regime shifts may be triggered by an exogenous shock, or they may occur through a slow-timescale erosion of resilience [35] (figure 1). In the latter case, a small incremental change or even just stochasticity or noise [36] can cause a regime shift, which may appear abrupt even though the system may have been approaching the tipping point for a long time [27].

For wide classes of regime shifts, new balancing feedbacks emerge that stabilize or lock the system in its new state [37,38] and make regime shifts difficult to reverse (hysteresis) [21,33]. While not all regime shifts exhibit irreversibility or hysteresis [39], the persistence of change is a common feature of social-ecological regime shifts [33]. For the purpose of this work, we restrict attention to regime shifts characterized by large, abrupt, nonlinear and persistent changes in systems driven by system-internal feedback mechanisms [33].

Regime shifts can occur in land systems [18,19], which are social-ecological systems comprising all activities, processes and outcomes related to human and environmental use of land [19,40]. Recent works have called for greater attention to land regime shifts and laid important theoretical groundwork. Ramankutty & Coomes [18] proposed a causal-mechanistic explanation of land regime shifts in terms of their preconditions, triggers and self-reinforcing feedbacks, qualitatively illustrating literature-based examples. Müller *et al*. [19] provide case-based qualitative and quantitative empirical evidence of land regime shifts, specifically identifying the rubber boom in northern Laos and the oil palm boom in Indonesia as examples of booms that constitute land regime shifts. All this suggests that a regime-shifts angle could offer unique insights about the dynamics of crop booms and their management [16].

The main aim of this article is to examine under what circumstances a crop boom constitutes, in fact, a land regime shift and to show that analysing crop booms as regime shifts affords insights about the mechanisms that cause the characteristic dynamics of booms and their lasting impacts. Our work proposes a middle-range theory [7] of crop booms as regime shifts. To do so, this novel lens on crop booms addresses several theoretical and empirical challenges.

First, the application of complex systems theory has resulted in sometimes vague and/or metaphorical use of concepts [33] and a low degree of specificity about the variables and mechanisms involved in tipping dynamics. Second, crop booms and regime shifts are inconsistently defined and the terms have been used interchangeably [19,41]. Third, there are few detailed case studies of crop booms combining quantitative land-use change trajectories with rich qualitative and quantitative descriptions of social-ecological dynamics within a broader political and institutional context (but refer to e.g. [19,42,43]), all of this being necessary for a social-ecological regime-shifts analysis.

Specifically, our work builds on existing literature to fill the following gaps: (i) Müller *et al*. [19] characterize certain crop booms as regime shifts but do not provide specific details about how exactly a crop boom is a regime shift other than exhibiting rapid and large changes; here, we propose a rigorous definition of crop booms and identify the overlaps and differences between crop booms and regime shifts. (ii) Ramankutty & Coomes [18] propose three specific causal mechanisms for land regime shifts (preconditions, triggers and self-reinforcing mechanisms), but do not mention crop booms; here, we characterize (land) regime shifts in terms of their dynamics (abrupt, rapid, profound and lasting change) and causal mechanisms (preconditions, triggers and self-reinforcing mechanisms) and apply this characterization to the analysis of crop boom dynamics. (iii) Castella *et al*. [1] discuss in detail the dynamics of crop booms, identifying characteristic patterns—booms-within-booms, waves of booms and variegated pathways—but do not apply a regime-shifts lens; here, we do just that to explain the dynamics of crop booms, including the dynamic patterns identified by Castella *et al*. [1].

Our work conducts an examination of crop booms as regime shifts based on the booms of rubber and banana in northern Lao PDR. The northern Laos rubber boom has been ascribed to the growing market for latex in neighbouring China, rising rubber prices, changes in land-use policies favouring cash crops and social influence within cross-border networks that spread information and new social norms to ‘get rich’ with rubber [44–46]. The banana boom was attributed to a combination of growing demand and pests decimating plantations in China, shifting production sites to northern Laos [47]. However, while these factors help explain the expansion of these cash crops in northern Laos, they do not account for the uneven spread in similar locations (but refer to [48] on geopolitical issues), nor do they provide an explanation for the central aspect of this article, namely the abruptness and nonlinearity, in other words, the ‘boomness’ of the expansion.

An earlier study examined the expansion of rubber in two study areas in northern Laos’ Luang Namtha Province [41]. It identified a number of market, policy and social preconditions, triggers and self-reinforcing mechanisms—notably, imitation—and showed that these helped explain, respectively, the occurrence, timing and intensity of rubber expansion in both areas. However, this earlier work did not explore in detail *how* imitation became self-reinforcing, it did not identify other self-reinforcing feedbacks and it did not explore the mechanisms linking the scale of causal mechanisms with the scale of expansion, nor did it assess how land regime shifts became difficult to reverse. Moreover, the earlier work did not attempt to provide a causal-mechanistic explanation of crop booms based on complex systems theory.

All of these gaps in our understanding of crop booms are addressed in the present work, which builds on these same empirical cases from northern Laos. We conduct additional quantitative and qualitative analyses and focus on two commodities, rubber and bananas, both of which exhibited boom-like dynamics in one study area and mild or no expansion in the other. We examine the trajectories of crop expansion during the period 2000–2018. Our analysis is based on empirical data collected in 110 semi-structured household surveys, focus groups in each village, as well as interviews with farmers, government officials and cash crop traders [41].

We first assess *whether rubber and banana expansion dynamics in the study areas meet the characteristics of a regime shift*, that is, an abrupt, rapid, nonlinear and permanent change caused by preconditions, triggers and self-reinforcing mechanisms, rigorously assessing our empirical data against each of these characteristics (§3). We then propose a *definition of crop booms based on complex systems theory* and our empirical evidence, identifying the causal mechanisms that explain the most important dynamic patterns of booms, constituting a *middle-range theory* [7] *of crop booms* (§4.3). As part of this, we examine the processes through which feedbacks become destabilizing (or not), illustrate how the same feedback (e.g. imitation) can induce boom-like versus milder expansion and analyse the connection between the scales at which mechanisms act and the scales at which a boom unfolds. Furthermore, we *disentangle the concepts of crop booms and regime shifts* (§4.1), examine the *role of resource frontiers* in booms and regime shifts (§4.2), discuss the main limitations (§4.5) and derive lessons learned for the *governance* of land systems prone to crop booms and land regime shifts (§4.4).

## 2. Material and methods

### 2.1. Study area description and land-use history

We focus on two case study areas in Luang Namtha Province in northern Laos, which we designate as Oudomsin (Sing District) and Prang (Vieng Poukha and Namtha Districts) after a village located in each [41]. Study areas were selected to be as similar as possible across socioecological variables while limiting the major difference to the variable of interest, namely the intensity of cash crop expansion (high in Oudomsin and low in Prang); such a combination of comparability and variation can be used in combined inductive, deductive and abductive approaches to formulate generalized knowledge claims about causal mechanisms [49].

Study site boundaries were defined to be of similar size (Oudomsin: 8.7 × 10.5 km, Prang: 9.5 × 12.1 km) and to encompass multiple villages [50]. Both study areas are considered agriculture–forest frontier areas [51] and comprise a mix of lowland and upland land uses and forested areas, including part of the Nam Ha National Protected Area (NPA). Prang is more mountainous and has a higher fraction of upland (sloping and higher altitude) areas (91% versus 80% in Oudomsin). Prang is also less densely populated than Oudomsin (0.18 persons ha^−1^ in Prang versus 0.61 in Oudomsin). In both areas, agricultural production is the predominant source of income, and farmers are smallholders with average holdings of 4 (±4) ha per household [52]. Before the widespread adoption of cash crops, agricultural production traditionally consisted of shifting cultivation of rice in upland plots, high-yielding irrigated rice production in lowland paddies and mixed production in flatland areas surrounding paddies.

The population in both regions belongs to minority ethnic groups, and affiliation differs between villages but is homogeneous within. Both sites are borderland regions, although Oudomsin has stronger cross-border interactions since it is located directly on the border with China and villagers have historically close kinship and social ties with villagers in China, especially within the same ethnic groups [53,54]. Conversely, villagers in Prang, located 60 km away from the border by highway, do not have such close cross-border social ties.

Both regions are connected by highway to Yunnan Province, China, but Sing District’s location at the border and abundant fertile land make it particularly suitable for the production of export-oriented cash crops for the Chinese market. Sing District experienced a boom in sugarcane production in the 1990s [55]. In the early 2000s, sugarcane production was fading as rubber plantations were expanding rapidly in parts of Luang Namtha Province caused by a steep and sustained rise in rubber market prices and new policies that favoured rubber production and trade [45,56]. Rubber expansion was boom-like in Oudomsin but slower and less intensive in Prang, and it continued in both areas during a period of sharply declining rubber prices after 2011 [50].

Banana plantations expanded in northern Laos around 2009 following weather- and pest-induced losses in China [47]. Farmers were offered lucrative leases (of the order of USD $2000 ha^−1^ yr^−1^) for contiguous rice paddies and surrounding flatland areas by (mostly Chinese) investors, who established and managed the banana plantations. Oudomsin was a preferred site for banana production, but Prang did not have suitable flatland areas. Banana plantations expanded very rapidly in Oudomsin but were discontinued after investors left after 2015, in part owing to new pest outbreaks.

At the beginning of the study period (year 2000), both regions could be considered natural resource frontiers with a large fraction of forest within and around village boundaries and a mixture of shifting cultivation, secondary forests of various ages and old forest in the uplands. Having undergone a previous sugarcane boom in lowland areas, Oudomsin was a more advanced frontier [57].

### 2.2. Data collection

#### 2.2.1. Household survey and interviews

A semi-structured household survey (*n* = 110) was conducted in 10 randomly selected households within six villages selected in Oudomsin (*n* = 60) and in all five villages in Prang (*n* = 50) in 2017–2018 [41]. All selected villages in Oudomsin belong to the same administrative cluster (kumban); in the Prang area, all four villages in Vieng Poukha District belong to the same cluster and one village in Namtha District belongs to a different cluster (see map of study area in [41,50]).

The household survey elicited quantitative information about household composition and plot-level land-use history since the 1990s or since the household was established and collected land-use data differentiating between lowland and upland land uses. Lowland uses include rice paddy, banana and sugarcane. Upland uses include forest, shifting cultivation fallow, upland rice, sugarcane, rubber and cardamom. The survey also asked open-ended questions about the reasons for planting each cash crop plot and how farmers first learned about the cash crops they planted.

Focus groups were conducted in each village (*n* = 11) to map history and significant events in time affecting major land-use transformations. We also conducted open-ended interviews with government officials at the district (*n* = 2) and province (*n* = 2) levels, as well as with private-sector agricultural investors and traders (*n* = 4), including three rubber companies [41].

#### 2.2.2. Other data

We use annual time-series land-use maps of each study area for the years 2000–2017 developed by Latthachack *et al*. [51] to assess the fraction of rubber in upland areas; we use these data because the household survey only captures land converted to a crop and excludes non-converted common land, such as forests. We obtained shapefiles of the Nam Ha NPA, which covers portions of both study areas, from the Sing District Office of National Resources and the Vieng Poukha District Area Forestry Office (DAFO). We compiled monthly rubber prices from the Singapore Commodities Exchange (SCE) for 1998–2017 [58].

### 2.3. Data analysis

We plot the cumulative fraction of adopters of rubber and banana and the fraction of each cash crop over the total upland (for rubber) or lowland (for banana) area in each study region for the period 2000–2018. We also plot the cumulative area of rubber and banana in each village during the same period.

To understand the causal mechanisms underlying land-use change, we analyse data from the household survey, interviews and focus groups, identifying social, market and policy factors influencing rubber and banana expansion dynamics at multiple scales. We examine the motivations for crop adoption explicitly mentioned by interviewees and identify exogenous changes (e.g. in policies or market conditions) that have a temporal correlation with land-use changes. We then analyse the match between our empirical data and the dynamics (rapid, large-scale and persistent change) and causal mechanisms (preconditions, triggers, self-reinforcing feedbacks and critical thresholds) of regime shifts [18,33]. A comparison of both regions allows us to gain additional insights about the operation of each causal mechanism.

We use an iterative combination of abduction, deduction and induction [59] based on qualitative and quantitative data in each case study area to formulate generalized knowledge claims [49] about crop boom dynamics. We propose a definition and causal-mechanistic characterization of crop booms (§4.3) based on our findings, other crop boom literature and other works that have examined nonlinear patterns of adoption and opinion dynamics.

## 3. Results: a complex systems perspective of rubber and banana expansion

Using a complex systems perspective, we examine here whether the expansion of rubber and banana plantations in the two study areas represents a land regime shift, i.e. a rapid, profound, nonlinear and persistent land system change [19], and whether the causal mechanisms that underlie regime shifts—preconditions, triggers and self-reinforcing mechanisms [18]—fit our data. We examine critical thresholds in §4.

### 3.1. Preconditions

Preconditions or predisposing factors refer to the configurations within and between systems that make a system prone to a regime shift [18]. Preconditions generally fall under three broad categories. First, a system may have gradually lost its resilience or may be operating near its critical threshold [32,60]; the *previous occurrence of a regime shift* can put the system in such a state of high criticality and create preconditions for subsequent regime shifts [18]. Second, a system may have *built-in self-reinforcing feedbacks* that may easily become destabilizing, such as a closely connected social group that is prone to imitation [61]. Third, high *connectivity* between systems can exacerbate positive feedbacks through contagion [24] or tipping cascades [62,63]. All three types of preconditions were present in Oudomsin and Prang, albeit with important differences.

#### 3.1.1. Previous booms predispose the system to criticality

In 2001, Oudomsin was in the midst of a sugarcane boom, which arguably primed the region for subsequent booms in various ways, as explained by villagers in interviews: many households switched to rubber when sugarcane required replanting after a 6-year cycle, households used cash savings from sugarcane to invest in rubber plantations and farmers had gained experience producing cash crops and doing business with international traders. Experiencing a boom possibly also led to a ‘get-rich-quick’ mentality [1,64]. Prang villagers had also experimented with other cash crops before the advent of rubber, but the region had not experienced a full-fledged boom.

#### 3.1.2. Built-in self-reinforcing feedbacks: within-village connectivity and imitation

Villages in Prang and Oudomsin constitute relatively small, homogeneous and tightly connected social units characterized by close cooperation. This may explain the high degree of social influence and imitation in land-use decision-making, particularly at the village level. The practice of planting in groups also gave rise to another built-in self-reinforcing mechanism, namely, neighbourhood effects (see §3.3).

#### 3.1.3. Connectivity with outside markets and social role models leads to contagion

Oudomsin and Prang are well connected with markets for agricultural commodities and inputs in Yunnan, with Oudomsin having a significant comparative advantage as a border region [65]. Yet, *social* connectivity, particularly between villages of the same ethnic group, probably played a more decisive role than physical connectivity in the spread of cash crops. When rubber prices started rising in 2001 and mature plantations in China became extremely profitable, Oudomsin villagers observed that their relatives across the border were ‘getting rich’, which became a powerful motivation for adoption [54]. Over 70% of Oudomsin households cited first-hand contact with Chinese villagers (mostly kin) who had ‘succeeded with rubber’ as the reason for adopting the crop [41]. In addition to spreading new values and aspirations for higher standards of living, cross-border social networks also served to exchange knowledge about rubber markets and production. In contrast to Oudomsin, Prang villagers mostly learned about the benefits of rubber from Lao and Chinese investors or in nearby villages, but obtained little direct evidence from close and trusted sources [41].

### 3.2. Triggers

**Figure 2.**
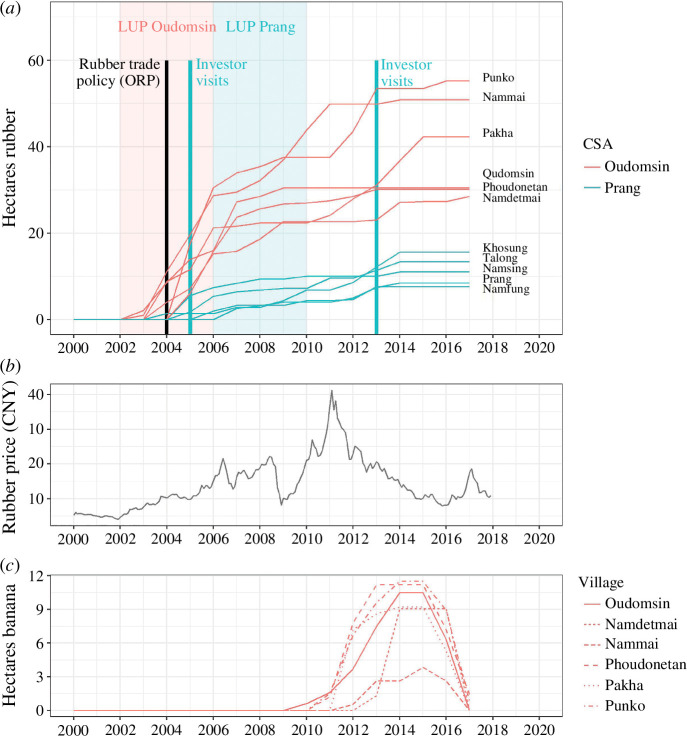
Triggers and trajectories of expansion. (*a*) Trajectories of rubber expansion in each village and case study area (CSA) (source: household survey), including triggers of adoption affecting both areas (black) or one of the areas (colour-coded). LUP = land-use planning. (*b*) Monthly rubber price in Chinese Yuan (CNY), SCE [58]. (*c*) Trajectories of banana expansion in Oudomsin area villages; the arrival of investors in the area after 2009 constituted the main trigger (not marked).

Triggers are rapid changes that can provide the immediate impulse for a regime shift [18] and help explain its timing [66]. In the context of farming, Sutherland *et al*. [67] argue that major triggers necessarily precede major decisions because the path-dependency, lock-ins and resilience of farming favour continuity instead of change. A shock, i.e. a drastic impact on the system caused by one or more sudden and strong perturbations, can act as a trigger, be it idiosyncratic (affecting individual households) or covariate (affecting many households at once, such as policy, environmental or economic changes) [18]. Often, it is not one large trigger, but rather a *confluence* of triggers acting in mutualistic or synergistic fashion—a conjuncture [68,69]—that provides the definitive impulse for a regime shift. In Oudomsin and Prang, we identify a confluence of major triggers for the expansion of cash crops (figure 2), namely, market and policy changes.

#### 3.2.1. Market changes

Rising rubber market prices after 2001 (figure 2*b*) constituted a major impulse for rubber adoption. Around 65% of households cited economic motivations for planting rubber. Villagers in Oudomsin and Prang were well-informed about market prices in China, with 76% of Prang farmers and 43% of Oudomsin farmers reporting that they knew the price at the time of adoption [41]. A 2004 rubber trade agreement (figure 2*a*), the Opium Replacement Policy, incentivized Chinese investments in Luang Namtha Province [70] and strengthened farmers’ expectations that a stable rubber market would ultimately develop in Laos. In Prang, visits from various rubber investors proposing rubber production contracts prompted many villagers to adopt rubber, with some villagers planting on an individual basis [41]. With regard to banana plantations, the arrival of Chinese investors offering land leasing contracts constituted the immediate impulse for the uptake of the crop and was itself a cascading result of pest- and weather-induced disruptions in China. Investors negotiated lease contracts with Oudomsin villagers in 2009 and with surrounding villages in the following years (figure 2*c*).

#### 3.2.2. Policy changes

In both areas, new land-use planning (LUP) regulations (implemented in 2002–2006 in Oudomsin and 2006–2010 in Prang) (figure 2*a*) significantly weakened land tenure of shifting cultivation land by declaring fallows older than 3 years unused, providing a strong incentive for planting a commercial crop such as rubber. LUP policies were similar in both areas, although enforcement was laxer in Prang [41].

### 3.3. Self-reinforcing feedbacks

A feedback is a process in which (some fraction of) the output is fed back into the input [71]. Generally, positive feedbacks have a self-reinforcing effect (reinforcing change in the direction of the deviation), while negative feedbacks are deviation-counteracting and have regulatory or equilibrating effects^1^ [71]. In the case of rubber and banana expansion in Laos, we identified three main feedbacks acting at different scales from plot to region, namely neighbourhood effects, imitation and a land rush.

#### 3.3.1. Neighbourhood effects (plot level)

The establishment of banana plantations in rice paddy areas thwarted rice production in adjacent land by dismantling irrigation channels, which hampered downstream rice production, and by exerting a push effect on rodent pests towards remaining paddies owing to heavy pesticide use in banana plantations. After some villagers had leased their paddies to banana investors, many others reluctantly followed suit after they could no longer grow rice. Neighbourhood effects also played a role in the expansion of rubber, as villagers could not carry out shifting cultivation of fallows adjacent to rubber fields for fear of causing accidental fires subject to costly compensations. Yet neighbourhood effects in the uplands were mitigated by the dispersal of rubber plantations and presumably had a smaller impact than in banana plantations clustered in the lowlands.

#### 3.3.2. Imitation (village level and between villages)

Around 40% of households in each study area cited ‘following the community’, meaning mostly members of one’s own village, but also nearby villages of the same ethnic group, as the main reason for adopting rubber [41]. Imitation was partly driven by normative considerations, such as reputational reasons (‘I don’t want others to think I am lazy’) and a desire to conform (‘I wanted to be like others’). An example of normative conformity is expressed in this adoption narrative: ‘(I planted rubber because) I want to have what others have; if rubber prices are good we all win, and if they are bad we all lose’. Imitation was an important motivation for adoption, although probably with different effects in Oudomsin and Prang. Oudomsin villagers aspired to own large rubber plantations like their relatives in China, and owning many rubber plots became a normative goal. As one Oudomsin villager stated: ‘I see others have a lot, so I also want a lot; if you have too little, then you are embarrassed’. In contrast, Prang households generally planted one or at most two rubber plots, possibly influenced by government guidance to plant one hectare, distrust of investors or because of their lower income.

#### 3.3.3. Land rush (village level and between villages)

In the earliest rubber boom stage, between 2003 and 2005, five out of six studied villages in Oudomsin cut down their communal forests and divided the land among many^2^ village households to plant rubber, despite the fact that this action constituted a severe infringement on land-use planning regulations [41]. These decisions were possibly inspired by villagers across the border in China, who had taken similar measures encouraged by government policies incentivizing rubber adoption [54]. The collective transformation of village forests into rubber plantations ensured an equitable distribution of benefits and reduced risks since government officials did not sanction the illegal deforestation ‘or everyone would have gone to jail’. The resulting rapid privatization of a large area (comprising around 40% of all rubber hectares by 2017) exacerbated competition for land within and between neighbouring villages. The combined effect of land scarcity and tenure insecurity constituted a powerful incentive to claim land by planting rubber lest someone else would do so earlier, and ‘everyone rushed to mark their plots’. The ensuing land rush was self-reinforcing, as planting rubber permanently reduced available land and increased pressure on the remaining areas. In contrast, the expansion of bananas did not appear to cause a land rush, presumably in part because production was concentrated in rice paddies, which—unlike fallows or forests—enjoyed clear ownership rights. In Prang, interview narratives do not point at the occurrence of a land rush, possibly owing to the lower population density in the region, the absence of collective village forest conversions and households’ smaller rubber plantations.

### 3.4. Profound and structural land system change

The expansion of rubber in Oudomsin transformed the uplands from mostly shifting cultivation land, secondary forests and old-growth forests to a rubber-dominated landscape. By 2017, rubber covered an estimated 10% of the NPA located within study area boundaries, 50% of protected village forests and 89% of shifting cultivation areas existing in 2001. Upland rice cultivation was all but abandoned. Forest loss negatively affected biodiversity and ecosystem services [73]. Changes in land tenure from customary (shifting cultivation) or collective (village forests) to private (rubber plantations) curtailed villagers’ access to hunting and wild food collection grounds [74]. The near-abandonment of shifting cultivation, requiring close cooperation between households, changed social and kin relations by reducing mutual dependence.

**Figure 3.**
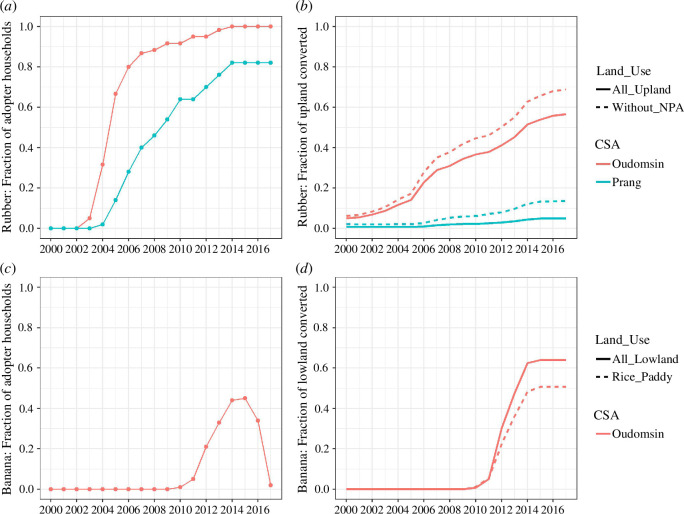
Trajectories of rubber (*a,b*) and banana (*c,d*) expansion in each case study area (CSA). (*a,c*) Fraction of adopter households. (*b*) Fraction of upland areas planted with rubber (including and excluding the NPA). (*d*) Fraction of all lowland and rice paddy areas converted to banana plantations and subsequent cash crops (mostly sugarcane). Data sources: (*a,c,d*) Household survey. (*b*) Land use maps [51].

In Oudomsin’s lowlands, traditional rice paddy terraces and irrigation channels were levelled to install hose irrigation systems for banana plantations. Household rice production and cultivated rice paddy area dropped by half (figure 3*d*), although households remained largely rice-sufficient [52]. Intensive use of pesticides polluted soil and river water and decimated fish stocks, an important source of protein. Leased paddies were no longer directly managed by their owners, who now had more time and cash to engage in other economic activities, not least planting more rubber. Sales of rubber latex and income from land leases doubled household income compared with 2005 levels [52]. The advent of cash crops also brought about durable changes in norms, values and attitudes towards money (see §3.6).

Our results thus suggest a profound and structural land change in Oudomsin. In Prang, the milder diffusion of rubber in upland areas had fewer associated social and ecological impacts: deforestation was substantially lower than in Oudomsin, households largely continued to practice shifting cultivation, and the amount of land converted to a cash crop (and associated social and ecological impacts) was relatively small. Unlike in Oudomsin, Prang villagers did not have a market opportunity to plant cash crops in rice paddies, and rice production was largely unaffected.

### 3.5. Nonlinear and S-shaped change trajectory

The cumulative adoption curve of rubber in Oudomsin is clearly nonlinear and follows an S-shape pattern (figure 3*a*) and shows that the crop underwent a period of very rapid expansion after the fraction of adopters reached 7% in 2003, only one year after initial uptake. Adoption reached 82% in 2006 and 100% in 2013, at which time rubber plantations covered over half of all upland areas and over 70% of upland areas excluding the NPA (figure 3*b*). The S-shape is less pronounced but still appreciable in Prang, where 82% of households had adopted rubber by 2014 after it was first planted in 2004, but rubber plantations comprised only 7.5% of upland areas by 2017 (13% excluding the NPA). The expansion of bananas in Oudomsin also follows an S-pattern, with a jump in uptake in the second year (2011), when the fraction of adopters was at 5% (figure 3*c*). Within three more years, almost 45% of households had leased one or more plots for banana production, and plantations extended over 60% of flatland and 50% of rice paddy areas (figure 3*d*). The bust that followed was even faster, and banana plantations disappeared within two years after 2015. The results show that the expansion of rubber and banana exhibits an S-shape pattern not only at the level of each study area but also within each village (figure *2a*,*c*).

### 3.6. Hysteresis and irreversibility

Hysteresis means that a system would need to follow a qualitatively different trajectory to return to its original condition [33], and this return is only possible if controlling variables are dialled down to a level far removed (on the safe side) from the original critical threshold [75]. Irreversibility means that recovery is no longer possible, no matter how far control parameters are dialled down [75], because the reconfiguration of feedbacks has locked the system in its new regime [38]. We identify a number of changes in Oudomsin and Prang that make a return to a pre-cash crop regime difficult and suggest hysteresis, namely land claims in rubber plantations, normative changes and the inability to restore previous crops.

#### 3.6.1. Planting a cash crop to claim land

Planting rubber became a means to secure and claim land [76] in the context of land tenure insecurity exacerbated by policy changes. Households could request land titles for rubber plantations, unlike for shifting cultivation or collectively owned forest land. Land titles are costly, time-consuming and grant the highest level of tenure security. Villagers have a high incentive to uphold land titles and are unlikely to revert to land-use practices that could put such titles at risk.

#### 3.6.2. Normative changes: new standard of living and ‘boom mentality’

The expansion of cash crops caused lasting transformations in the imaginaries, norms and values of the population. Oudomsin villagers used the large and unprecedented influx of cash to build brick houses and replace traditional bamboo and wooden structures, purchase motor vehicles, reinvest in rubber plantations and pay for their children’s education. Living standards closer to those of their relatives in China became the new normal, and narratives expressing a wish to ‘get rich’, uncommon in Laos prior to the expansion of rubber [54], became prevalent. The succession of cash crop booms probably cemented the ‘boom mentality’ of anticipation of new booms [1]. Villagers who fully or partly abandoned rice production newly saw themselves as entrepreneurs rather than as subsistence farmers [54,77], and many expressed unwillingness to return to labour-intensive and low-yielding shifting cultivation practices.

#### 3.6.3. Inability to restore previous crop

Farmers who wanted to reinstate rice paddies in abandoned banana plantations in Oudomsin did not have the machinery (tractors) or financial means necessary to uproot banana trees and restore terraces and irrigation channels. The problems that had traditionally plagued paddy rice production—poor irrigation infrastructure and unreliable yields—added to farmers’ reluctance, and many decided to wait for a long-promised government irrigation project before investing in paddy restoration. Most farmers switched from banana to sugarcane instead, a shift that was facilitated by a sugarcane company offering land preparation in return for contract farming arrangements [1]. Many continued to lease out their plots, mostly to nearby villagers, rather than resuming the direct management of their land.

### 3.7. A regime shift?

In Oudomsin, the expansion of rubber in the uplands and banana in the lowlands each appear to meet the characteristics of a regime shift (and a boom; see §4), since each brought about major and lasting social and ecological changes (table 1). These changes made it increasingly difficult to return to subsistence-centred farming even when market conditions returned to pre-boom levels, such as during the decline of rubber market prices or the collapse of the banana market. The succession of the sugarcane, rubber and banana booms can also be understood as stages of a larger regime shift from subsistence to commercial agriculture (figure 1). Conversely, the milder expansion of cash crops in Prang does not clearly qualify as a regime shift or a boom. While cumulative adoption curves of rubber exhibit an S-shape, the fraction of converted land was relatively small, causing correspondingly fewer impacts and arguably no fundamental restructuring of the land system.

**Table 1.** Does the expansion of rubber and banana in the study regions meet the definition of a (i) crop boom and a (ii) land regime shift?

characteristic	**definition**	rubber, Prang	rubber, Oudomsin	banana, Oudomsin
**crop boom**				
temporal pattern	rapid, nonlinear expansion that follows S-shape pattern	adopter fraction: yes (figure 3*a*) fraction of land: no (figure 3*b*)	yes (figure *3a*,*b*)	yes (figure *3c*,*d*)
causal mechanism	social self-reinforcing mechanisms	imitation; neighbourhood effects (weak)	imitation; land rush; neighbourhood effects (weak)	imitation likely but no empirical evidence gathered; strong neighbourhood effects
spatial scale	scale of boom relates to scale of self-reinforcing mechanisms	village-level (imitation); plot-level (neighbourhood effects)	village-level (imitation, land rush); plot-level (neighbourhood effects)	within-village (imitation); plot-level (neighbourhood effects)
crop boom?	all of the above	no?	yes	yes
**land regime shift**				
preconditions	predisposing factors [66] that provide the necessary conditions for the regime shift to occur [18]	60 km to Chinese border; cross-border physical connectivity (highway)	3 km to Chinese border; strong cross-border physical connectivity (highway, proximity); cross-border social connectivity; earlier booms	physical connectivity with China (highway, proximity); earlier booms; inadequate paddy irrigation infrastructure
triggers	rapid changes that provide the immediate impulse for a regime shift [18] and help explain its timing [66]; often a confluence of multiple triggers acting in mutualistic fashion [33]	rubber price increases; new land-use regulations; traders visit the area	rubber price increases; new land-use regulations	banana investors offer large lease amounts
tipping point	critical threshold beyond which system-internal feedbacks operating at different scales destabilize the system [33]	no tipping point?	critical fraction of land converted to rubber; critical mass of adopters; exacerbated by land scarcity and land rush	critical fraction of rice paddies converted to banana
self-reinforcing feedbacks	positive feedbacks [33,78] can be stabilizing, maintaining the new regime and establishing conditions that make it difficult to shift back to the old regime [18]. Beyond a critical threshold, they have a destabilizing effect and bring about a regime shift [21].	within-village imitation less destabilizing than in Oudomsin because households aspire to ‘only’ 1–2 ha of rubber and not ‘as much possible’; weak neighbourhood effects (risk of burning adjacent rubber plantations); no land rush	within-village imitation accelerates adoption beyond critical threshold; new norms (becoming rich with rubber and owning large plantations) exacerbate imitation and land rush; weak neighbourhood effects (risk of burning adjacent rubber plantations); land rush within and between villages	within-village imitation accelerates adoption beyond critical threshold; strong neighbourhood effects (banana plots damage downstream irrigation and push pests out to neighbouring plots); no signs of land rush
hysteresis or irreversibility	a return to the pre-regime shift state is only possible if controlling variables are dialled down to a level far beyond the original critical threshold; for irreversible changes, recovery is not possible [75]	same as in Oudomsin; however, the impact is lower owing to the lower extent of rubber expansion	getting used to higher standard of living; ‘boom mentality’; land titles for rubber plots (and no titles for shifting cultivation or forest) durably change land tenure and access	getting used to higher standard of living; ‘boom mentality’; reverting to rice paddy after planting bananas requires high capital investment; lease arrangements persist after banana bust
path dependencies	the present state is a function of (influenced by) past states		boom → boom (here: sugarcane → rubber)	paddy → banana → sugarcane
multiple scales	social and ecological dynamics take place at various spatial and temporal scales and can interact and amplify each other across scales	neighbouring plots; within-village (imitation, collective action, land scarcity); village clusters (imitation, land scarcity); cross-border connectivity	same	same
cascades and contagion across sites	a regime shift can influence the occurrence of another regime shift through cascading effects [62] or contagion [79,80]	rubber expands in China → then Laos	rubber expands in China → then Laos	banana expansion in China followed by pest outbreaks → banana expansion in Laos followed by pest outbreaks
land regime shift?	abrupt, large and persistent change in the structure and functioning of a (land) system	no?	yes	yes

Underlined elements denote the main differences between rubber expansion in Prang and Oudomsin.

## 4. Discussion

In a recent work making an important contribution to our understanding of crop booms, Castella *et al*. [1] highlight three regular features of crop booms, namely that they are nested within each other; follow a pattern of emplacement, displacement and replacement; and are subject to diverging trajectories and variegated outcomes despite similar starting conditions. Here, we use a complex systems lens to develop theoretical explanations for the patterns identified by Castella *et al*. [1], along with other features of booms, including their intensity, nonlinearity and persistence.

Oudomsin’s favourable geographic and socioeconomic conditions and greater proximity to markets make it a more suitable candidate than Prang for the expansion of commodity crops. However, we argue that analytical approaches based on gradual change are not able to capture the *sudden intensity* of such expansion. We have suggested that the nonlinearity of expansion is attributable to self-reinforcing feedbacks operating at multiple scales. In §4.3, we highlight that these feedbacks were social in nature and propose that multi-scale social feedbacks are an essential feature of crop booms that explain their intensity and S-shaped pattern. As we discuss below, feedbacks were slightly different in Oudomsin and Prang. Neighbourhood effects and imitation were pre-existing, or *built-in*, in both regions even before the expansion of cash crops, but a land rush emerged in Oudomsin as crop expansion unfolded. Imitation, affecting not only *if* but also *how* innovations are adopted, had a more destabilizing effect in Oudomsin.

A focus on economic and geographic factors as the main drivers of land-use change [81,82] in crop booms [83,84] misses other aspects highlighted in this work, namely that market factors acted not so much as drivers but as initial *triggers* of adoption [50] and later lost in significance, with expansion becoming seemingly decoupled from market factors owing to path dependencies. Changes in policies were similarly important triggers for these and other booms [85–89], and previous booms paved the way for subsequent ones [90]. Despite being exposed to similar triggers, Oudomsin and Prang followed different trajectories and represent an example of variegated pathways and impacts of agrarian change [1], attributable in part to differences in endogenous preconditions and feedbacks.

Next, we examine the overlaps and differences between crop booms and regime shifts (§4.1) and the role of resource frontiers in both (§4.2). We propose a definition and causal-mechanistic explanation of crop booms based on complex systems thinking (§4.3) and discuss the governance implications (§4.4) and finally the limitations of our approach (§4.5).

### 4.1. Are all crop booms regime shifts?

Crop booms, narrowly defined, refer to a change in land cover. A land regime shift refers to a broader social and ecological restructuring of the land system. Crop booms and land regime shifts can take place in the absence of each other. Land regime shifts can occur in many forms and are frequently not associated with a crop boom. Examples include forest transitions driven by self-reinforcing out-migration [20] or structural large-scale land abandonment following political collapse [91].

Crop booms can theoretically take place in the absence of a land regime shift if the transformation is not major and persistent, without new feedbacks forming. Yet, most booms seem to have profound and lasting impacts and involve a fundamental restructuring of many aspects of the land system, such as ecosystem services, land ownership and production structures, land rents, resource access, distributive mechanisms, social relations and local culture [5,92,93]. That is, most crop booms appear to occur as part of, and in conjunction with, a land regime shift. This is particularly the case in frontiers, where the transition from extensive land uses to intensive commodity production almost inevitably involves such structural changes.

### 4.2. What is special about frontiers?

Many resource booms have occurred in natural resource frontiers^3^ [1,10,13,15,69,97], where the potential for resource extraction is (much) larger than actual extraction rates [57]. On one hand, numerous lock-ins and stabilizing feedbacks keep the system in a low-intensity, pre-frontier regime [98,99].

On the other hand, sudden changes that significantly facilitate resource extraction, improve market access or increase profitability—such as infrastructure projects, commodity price spikes, technological development or regulatory changes [18,100,101]—can cause a *large and abrupt* increase in the return to land or expectations thereof. The resulting real or anticipated *land rent gap* [100] creates a large incentive for the intensive use of land; in the words of Tania Li, it ‘makes land valuable in a spectacularly new way’ [102].

While these dynamics are not exclusive to natural resource frontiers, frontiers might be particularly susceptible to resource booms because their inherently large natural resource appropriation gap makes them highly sensitive to changes in external conditions. Insecure land tenure, a hallmark of frontiers [13], may exacerbate this effect by reducing the incentive to maintain stocks (‘use it or lose it’) [16].

### 4.3. Definition and causal explanation of crop booms from a complex systems perspective

**Figure 4.**
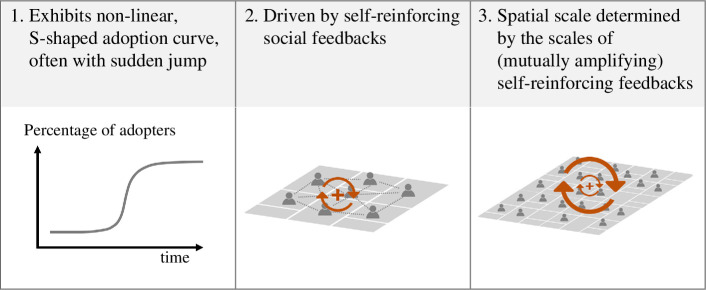
Definition of crop booms.

Based on our analysis of crop booms—those described in this work and other cases presented in the literature—we propose that crop booms are characterized by a set of regularly occurring features and underlying causal mechanisms (§4.3.1–4.3.4), which are necessary elements in a rigorous definition of a crop boom that we propose here: *crop booms are localized instances of sudden and intense expansion of a new commercial crop, with nonlinear cumulative adoption curves that resemble an S-shape, driven by predominantly social self-reinforcing feedbacks that operate at multiple scales* (figure 4).

#### 4.3.1. Preconditions, triggers and self-reinforcing feedbacks explain the ‘why’, ‘when’ and S-shape (‘how’) of booms

**Figure 5.**
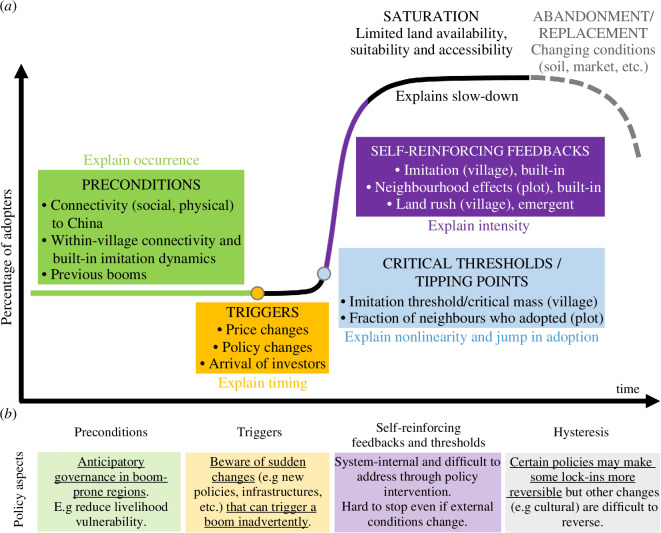
(*a*) Causal-mechanistic explanation of crop boom dynamics based on complex systems theory. Examples of preconditions, triggers, self-reinforcing feedbacks and critical thresholds are based on empirical evidence of rubber and banana expansion in the study areas. (*b*) Policy-relevant aspects; leverage points for management interventions are underlined.

Preconditions (e.g. social, market, biophysical and/or institutional) explain the local occurrence of a boom. Triggers—most likely a confluence of multiple and mutually reinforcing triggers—explain booms’ suddenness and timing. The phase of rapid, nonlinear, intense and generally S-shaped expansion is mainly attributable to internal self-reinforcing feedbacks that destabilize the system beyond critical thresholds (figure 5*a*). That is, preconditions, triggers and self-reinforcing feedbacks explain, respectively, the ‘why’, ‘when’ and intensity of booms. Among these, feedbacks are the key mechanisms that shape how booms unfold. The mere presence of feedbacks does not imply that a boom or a regime shift will take place; in fact, when the system is far from its tipping point (critical threshold), feedbacks have a stabilizing effect. In the rest of this section, we discuss important details about how feedbacks operate in the context of crop booms.

#### 4.3.2. Types of feedbacks and how they become destabilizing beyond a critical threshold

The literature on regime shifts highlights two distinct pathways through which feedbacks become destabilizing beyond a critical threshold: (i) the same feedbacks switch from stabilizing to destabilizing [21,22,24], or (ii) there is a change in the overall mix of stabilizing and destabilizing feedbacks in favour of the latter [16]. Our empirical analysis suggests that both pathways were present. Moreover, our analysis suggests that the former were pre-existing, *built-in* feedbacks, and the latter were new feedbacks, which we term *emergent*.

##### 4.3.2.1. Built-in feedbacks

Imitation and neighbourhood effects were built-in (i.e. pre-existing) self-reinforcing (i.e. positive) feedbacks in both study areas. Positive feedbacks inhibit the diffusion of an innovation when it is rare but encourage it beyond a certain take-off point [22]. This aspect is important: positive feedbacks do not *per se* have a destabilizing effect under all circumstances, but only after a certain critical threshold is exceeded; below that threshold, they have a stabilizing effect. In the study regions, imitation and neighbourhood effects stabilized the subsistence regime when adoption of the new cash crop was still low, but the same feedbacks tipped the system towards cash crop dominance (figure 1) once certain critical thresholds were exceeded (figure 5a).

Plot-level neighbourhood effects arose from the practice of planting subsistence and commodity crops by groups of households in spatial clusters. The critical threshold operating in this case may be the fraction of adjacent or nearby plots converted to a new crop below which continuity is favoured and above which conversion is incentivized (e.g. because growing the previous crop is no longer feasible). For imitation, the threshold may be a critical mass of adopters within the village or even adoption decisions by specific individuals with high prestige, both of which induce others to follow suit. Both conformist and prestige bias are forms of biased imitation that lead to S-shaped adoption patterns [22].

Importantly, our data suggest that the destabilizing effect of imitation was not only related to the decision of *whether* to adopt the new crop but also to the decision of *how much* to adopt—a fact that is often overlooked in innovation adoption and diffusion models that incorporate social influence [103,104]. If the norm is to allocate a small fraction of land to the new crop and there is no in-migration, then a commodity may still exhibit S-like expansion without a full-fledged boom or regime shift, as was the case in Prang. If the norm is to plant ‘as much as possible’, as was the case in Oudomsin, the result is not only S-shaped land-use change dynamics but also large-scale and boom-like conversion that targets all remaining land and can additionally lead to land-rush dynamics.

##### 4.3.2.2. Emergent feedbacks

The land rush in Oudomsin unfolded as an additional and new self-reinforcing feedback as crop expansion proceeded rapidly and used up seemingly all available land. The land rush was thus not built-in from the beginning but rather emerged as a result of other self-reinforcing mechanisms in combination with land scarcity. Hence, it appears to follow the second type of pathway. Here, the critical threshold may be the fraction of untitled land within and around villages converted to a cash crop that activated and exacerbated villagers’ perception of land scarcity, inducing even more people to claim the remaining land by planting a cash crop [105,106].

### 4.3.3. The main self-reinforcing feedbacks are social in nature

Neighbourhood effects, imitation and the land rush were all social in nature: they involved *relational mechanisms* such as social influence and normative conformity in imitation; sociotechnical *proximity mechanisms* organizing interactions in time and space [107] such as land-use planning or clustered agricultural activities giving rise to neighbourhood effects; or both in the case of the land rush. We posit that the main self-reinforcing mechanisms acting in the context of crop booms are social in nature (figure 4).

Beyond social influence [10,54,68,108] and land rush dynamics [64,68,109], other self-reinforcing mechanisms have been cited in the context of agricultural expansion. These include agglomeration economies [98,110]; policies that stimulate the emergence of powerful actors and rural elites, who lobby for more favourable regulations [100,111]; in-migration of farmers attracting more migrants through network and herd effects [13 20,112,113]; or reinvestment of high profits [18]. Strikingly, all except the last are social in nature. Following our definition of crop booms, agricultural expansion fuelled by fully independent profit reinvestment decisions alone would not constitute a boom, but it would also be highly unlikely, as farming decisions are affected by multiple factors [114,115].

As a corollary to this point, the necessarily social nature of booms implies that agricultural expansion caused by one independent agent, such as a single agribusiness corporation with a very large land concession, would not qualify in itself as a boom. However, such a large-scale land acquisition might become a potential triggering event for a land regime shift [116].

### 4.3.4. Self-reinforcing feedbacks operate at multiple scales and can amplify each other

The scale at which feedbacks affect dynamics is the scale of the interactions involved in those feedbacks. For example, social feedbacks operate at scales associated with the structures affecting agents’ interactions, such as infrastructure and institutions [117]. Thus, the scale of a crop boom is determined by the scale of the feedbacks involved (figure 4). Moreover, self-reinforcing feedbacks operating at different scales can amplify and feed into each other [16]. We suggest that the existence of feedbacks operating at different scales and possibly amplifying each other partly explains two well-known aspects of booms, namely booms within booms and sudden jumps in adoption. Moreover, diffusion mechanisms at broader scales help explain waves of booms across larger geographic regions.

#### 4.3.4.1. Booms within booms

It has been recognized that crop booms are likely to emerge as booms within booms at various spatial scales [1]. We suggest that each boom–scale combination corresponds to the scale of a certain self-reinforcing dynamic. In our analysis, adoption trajectory plots show S-shaped expansion of rubber and banana in the Oudomsin area, as well as in each village studied within that area. Rubber follows a similar pattern in Prang. This finding supports Castella *et al*.’s [1] assertion that booms are nested within booms—in this case taking place at village and higher levels. Our findings also suggest that feedbacks at different scales amplified each other because adoption decisions among neighbouring plots affected the fraction of adopters at the village level and vice versa, culminating in a self-reinforcing rush to convert untitled land within and around villages, which in turn further exacerbated neighbourhood effects and imitation processes. In other words, self-reinforcing feedbacks acting at different scales can amplify one another and have a cumulative impact that is larger than the sum of the parts.

#### 4.3.4.2. Sudden jumps in adoption

The rate of cash crop adoption in the study regions jumped abruptly from low to high, a feature that is appreciable in other empirical S-curves of agricultural innovation [118–120]. In Oudomsin, the jump lies at around 8% for rubber and 5% for banana (figure *3a,c*), which is at the lower end of reported group sizes of 10–40% in social tipping processes [26,117]. We postulate that the low jump threshold in Oudomsin might be related to the mutually amplifying effect of cross-scale feedbacks discussed above. Another explanation for low jump thresholds is a context of high uncertainty [24], such as that caused by tenure insecurity, volatile markets and weather shocks in Laos. Finally, it is important to take into account that jumps in adoption should be understood as the aggregate result of feedbacks and dynamics operating at various scales within a given window of analysis, rather than as a tipping point representative of a region or process type; the choice of a different spatial window of analysis is likely to result in a different jump threshold.

#### 4.3.4.3. Waves of booms

Beyond the scales at which feedbacks operate, booms can spread between socially and physically connected locations through cascading effects [62] or contagion [79,121], which may explain the waves of booms sweeping through Southeast Asia in the last decades [1]. Levin’s [122] assertion that different mechanisms explain patterns at different scales applies here as well: boom patterns appear to be driven by diffusion mechanisms on broad scales and are autonomously generated by intrinsic feedback mechanisms on fine scales.

### 4.4. Governance implications

Crop booms and regime shifts pose a challenge for management: they are abrupt and hard to predict; can cause high social, environmental and economic costs, and can increase social and ecological vulnerability [16]. Their speed makes adaptation difficult and exacerbates their impacts. They can be triggered by small changes that, in other circumstances or contexts, may not induce a critical transition. Self-reinforcing feedbacks responsible for rapid and nonlinear change are difficult to stop, and the resulting reconfiguration can lock the system in a new state [16]. Such dynamics call for analytical instruments and governance approaches that are different from those in place to address incremental change [17,18].

#### 4.4.1. Difficult to recognize early but can be anticipated

Crop booms are almost by definition not identifiable as such at their outset, but only after land-cover changes have unfolded significantly. At this point, the tipping process has probably been activated and feedback mechanisms driving the transformation at various scales have become destabilizing and are difficult to stop. The difficulty to identify booms early and their unpredictability [19] contribute to the frequently reactive nature of policy responses [1]. Early warning indicators such as increased autocorrelation or variance [60,123] can in theory signal dynamics that are approaching criticality, but measuring social tipping presents important practical challenges [26].

An alternative to early recognition is to identify land systems that are prone to regime shifts, i.e. to anticipate booms. Knowing that booms can arise rapidly following initial adoption—particularly in frontiers and/or in localities having undergone previous booms [90] and presenting high biophysical and social connectivity [124]—can allow policymakers and jurisdictions to prepare for eventual booms, including scenario planning, stakeholder engagement and implementing pre-emptive measures. Such anticipatory governance targets management interventions while they are still possible [125] (figure 5*b*).

For example, Sing District could have followed through with the promised irrigation infrastructure project, which would have enhanced the resilience of agricultural production and could have prevented or slowed down the expansion of banana plantations on valuable rice paddy land. Measures aimed at reducing livelihood vulnerability, such as affordable healthcare and education, farm insurance or low-interest loans that do not use agricultural land as collateral [52], may also make farmers less prone to adopting commodities in a boom-like fashion.

#### 4.4.2. Hard to stop contagion-like spread

Once self-reinforcing dynamics set in, they become the dominant force driving the system. This helps explain why land-use decisions may become decoupled from original triggers such as market prices [50]. Also, when the dominant dynamic driving land change is multi-scale contagion fuelled by self-reinforcing mechanisms, optimal site selection based on biophysical suitability loses importance as a criterion for land-use decisions. All this explains why booms are hard to model. From a governance perspective, understanding self-reinforcing dynamics is not only essential to anticipate how crop booms might unfold but also serves as a precautionary tale: policy and government interventions such as building a dam or a road or making large changes in policies [126] may induce a sudden land rent gap and trigger destabilizing and unanticipated processes—including, possibly, a crop boom—that are difficult to stop (figure 5b).

#### 4.4.3. Window of analysis may miss patterns of change

Pattern is inextricably linked to the window of analysis: drawing too large or too small a window might miss dynamics if there is a mismatch between the size of the window and the scale at which relevant feedbacks operate [122]. A large window of analysis at the national or province level may suggest that a crop is undergoing steady or linear expansion, while a lower-resolution analysis may show consecutive local booms in different locations [1]. Capturing booms in land-use change data thus requires fine-tuning the scale of analysis and paying close attention to local dynamics.

#### 4.4.4. Scale mismatches imply feedback mismatches

Scales at which natural resources are governed often do not match with the scale of environmental and social dynamics [127]. In Oudomsin, individual fines were ineffective against collective deforestation decisions, and collective sanctions or incentives may have been more effective in preserving village forests. Temporal mismatches [30] caused a delay in some stabilizing feedbacks such as pest outbreaks, but information transfer in social networks was rapid enough to prevent some farmers from adopting banana after learning about its long-term negative outcomes. Jurisdictions, too, can play an important role in the dissemination of information.

#### 4.4.5. Hysteresis, path-dependency and lock-ins

Certain social-ecological changes could be made more reversible through management interventions that remove lock-ins (e.g. providing economic support to rebuild irrigation infrastructure) or reduce hysteresis. For example, granting titles for shifting cultivation fallows would allow farmers to more easily switch back and forth between shifting cultivation and cash crops. However, in the aftermath of a crop boom, some changes are likely to persist even with such interventions, as stakeholders tend to reinvent local rules and relations in line with the new emerging social-ecological system [1].

### 4.5. Limitations

Our approach to identifying preconditions, triggers and self-reinforcing mechanisms is based on qualitative narrative analysis combined with quantitative land-use change trajectory data obtained from the household survey. Our methodology is not intended—nor does it allow us—to quantitatively assess the relative strength of various self-reinforcing mechanisms or triggers or to quantitatively differentiate between feedback types. Rather, we jointly interpret qualitative and quantitative data and use a combination of inductive and deductive thinking, which allows us to identify and understand causal mechanisms [49] and to propose the theory outlined in §4.3. Future work could numerically assess the relative importance and sequence of the different causal mechanisms, e.g. by using modelling approaches that quantify contagion and cascading dynamics [62,80].

The cases of rubber and banana expansion in Laos are paradigmatic examples of crop booms and bear many similarities to other booms in Southeast and East Asia [1], many of which were driven by similar exogenous factors such as high global demand for commodities and land-use reforms imposed by foreign donors consisting of land-use planning, delimitation of conservation areas and interdiction of shifting cultivation. Booms occurring in different contexts, such as in Europe [9] or in North America [8], also appear to bear striking resemblances in their internal dynamics; that is, they all seem to be fuelled by mainly social self-reinforcing mechanisms. However, additional detailed analyses of booms in different biogeographical and socio-political settings and involving various constellations of land users, power dynamics, commodity types, etc., are required to verify the broad validity of the crop boom definition and causal-mechanistic explanation proposed herein.

## 5. Conclusion

The dynamics of crop booms and land regime shifts—abruptness, speed, nonlinearity and large-scale change—present important analytical and governance challenges. Conventional logic suggests that market forces and biogeographical characteristics are the most important factors influencing the emergence of booms. In this work, we apply a complex systems perspective, focusing on regime shifts, to provide new insights about crop boom dynamics. We propose that triggers such as market and policy changes explain the timing of crop booms, and preconditions explain their local occurrence but not their intensity, nonlinearity and ‘runaway reaction’-like nature. Rather, we attribute the last features to self-reinforcing feedbacks, which were social in nature. The presence of positive feedbacks operating at multiple scales and sometimes amplifying one another can help explain well-known characteristics of booms, such as the nested occurrence of booms within booms or sudden jumps in adoption. Based on these insights, we propose a definition and causal-mechanistic explanation—a middle-range theory—of crop booms and identify possible leverage points for management interventions, such as implementing pre-emptive measures that increase social-ecological resilience, considering the trigger potential of sudden policy changes or reducing lock-ins.

Our novel perspective of crop booms as regime shifts in land systems connects two, so far disconnected, fields of research and can yield valuable insights into the analysis of other sudden, rapid and lasting land-use changes. Our work is informed by case study research in the context of smallholder agriculture in a forest frontier setting, and it remains to be assessed to what extent it is valid in other contexts. Further knowledge gaps that could be addressed in future empirical work include, for example, the identification of early warning signals of social or biophysical nature, the relative role and strength of different feedback mechanisms, the existence and numerical value of critical thresholds in different contexts or the factors exacerbating or diminishing lock-ins, hysteresis and path-dependence.

## Data Availability

We have included all the data used to develop figures 2 and 3 as supplementary materials [128].

## Footnotes

1 In rare cases, the opposite can be true, for example, in specific instances of delayed feedback in oscillatory systems [72].

2 In focus groups, village chiefs reported that village forests were supposed to be divided among all village households; yet, household surveys and interviews revealed that some households in each village were left out of this land apportionment.

3 The concept of frontier is contested and multi-layered [94–96]; here, we use one relatively widespread definition.
